# Exploring the Feasibility of a Generic Early Intervention Programme for Emerging Personality Disorder in Adolescents: Guideline‐Informed Treatment for Personality Disorders—Adolescent Version (GITPD‐A)

**DOI:** 10.1002/pmh.70109

**Published:** 2026-10-21

**Authors:** Paulien Jansen, Jacquelijne Schraven, Sarah Laschen, Stephan Kraai, Joost Hutsebaut

**Affiliations:** ^1^ NL‐PSY Zwolle the Netherlands; ^2^ Altrecht Child and Youth Psychiatry Utrecht the Netherlands; ^3^ iHub Amsterdam the Netherlands; ^4^ Karakter Child and Youth Psychiatry Nijmegen the Netherlands; ^5^ Viersprong Institute for Studies on Personality Disorders Halsteren the Netherlands; ^6^ Department of Medical and Clinical Psychology Tilburg University Tilburg the Netherlands

## Abstract

The Guideline‐Informed Treatment for Personality Disorders—Adolescent version (GITPD‐A) is an accessible, general approach to treating personality disorders (PDs) in adolescents. This study assessed the feasibility of the intervention and of a multicentre research design, as a preliminary step towards larger, better‐controlled trials. A one‐group pretest–posttest design was used across five mental healthcare institutions. Eligible participants were adolescents aged 12–18 meeting threshold criteria for a PD under the DSM‐5 alternative model. Participants (91.9% female; mean age 15.9 years) received GITPD‐A for 6 months. Intervention feasibility was evaluated via paired‐samples *t*‐tests on pre‐ and posttest scores. Feasibility of the multicentre design was assessed by eligibility and enrolment rates, measure completion and study dropout. Significant improvement was found for pathological personality traits (PID‐5), with a medium effect size (Hedges' *g* = 0.50). Secondary outcomes showed significant reductions in depression, anxiety, emotion dysregulation and symptoms of borderline PD. Participants reported high satisfaction (*M* = 8.1). Following a broad screening process involving 245 participants, 65 were found to be eligible. Of these, 47 adolescents took part in the study overall, with 38 completing the intervention. The treatment dropout rate was 19.1% (*n* = 9). Despite the limited sample, preliminary findings support the feasibility of GITPD‐A for adolescents with emerging PD, showing high acceptability, satisfaction, retention and acceptance of scientific assessments. Selected measures appear suitable for detecting intervention‐related changes. However, a different primary outcome and improved screening strategies may be needed.

## 1 Introduction

Personality disorders (PDs) are a major worldwide mental health problem due to their high prevalence (Tyrer et al. 2015), significant functional impairment (Chanen et al. 2007), lifelong disturbances (Biskin 2015) and substantial treatment costs (Bender et al. 2001). Early symptoms of PDs often first appear during adolescence, and most adults with PDs first sought help during their teens (Fonagy et al. 2015). However, PDs are often overlooked in young people in regular clinical practice, and PD impairments are usually inadequately treated or addressed too late (Bozzatello et al. 2019; Chanen 2015; Laurenssen et al. 2014). Consequently, personality problems may become chronic and greatly impact the psychosocial development of adolescents (Chanen 2015; Hastrup et al. 2019). Early detection can lead to earlier interventions, preventing the development of inflexible, maladaptive and chronic pathological personality traits that could otherwise result in significant functional impairment in adulthood (Hutsebaut, Clarke, and Chanen 2023).

Over the past decade, a limited number of randomized studies have supported the feasibility of specialized early intervention programmes for early signs of borderline PD (Jørgensen et al. 2021). These programmes include mentalization‐based treatment (MBT) (Rossouw and Fonagy 2012) and dialectical behaviour therapy (DBT) (Mehlum et al. 2014), both of which have proven effective in treating PDs in adolescents. However, the capacity for evidence‐based specialist psychotherapy is insufficient to meet the needs of these young people. Interestingly, recent studies and meta‐analyses of adult BPD patient samples support the effectiveness of generalist programmes such as Structured Clinical Management (SCM; Bateman and Krawitz 2013), Good Psychiatric Management (GPM; Gunderson and Links 2014) and Good Clinical Care (GCC; Chanen et al. 2008) for adults with BPD (e.g., Cristea et al. 2017; Storebø et al. 2020). Unlike specialist treatments, generalist programmes are not theory‐driven or prescriptive in terms of the setting, modalities, or content of sessions. Instead, they are typically more pragmatic, based on general principles, and focused on common effective factors. This makes them easier to apply across different types of PD and in less specialist treatment services. The Guideline‐Informed Treatment for Personality Disorders (GIT‐PD) is a Dutch generalist, common‐factor approach to treating PDs (Hutsebaut et al. 2020; Hutsebaut, Kindt, et al. 2023; Hutsebaut, Videler, et al. 2023). GIT‐PD offers a basic framework based on common features of evidence‐based treatment approaches for PDs. GIT‐PD emphasizes that treatment should be time‐structured, therapists should adhere to common factors, and treatment should be embedded in a team and organizational context (Hutsebaut et al. 2020). Recently, GIT‐PD was adapted for young people with emerging PD and their families. GITPD‐A is designed for young people (and their families) with complex emotional needs who are at high risk of developing serious mental health issues, especially PDs. FR The primary aim of GITPD‐A is to prevent personality problems from significantly impacting adolescent development. To achieve this, it designs a pathway for early detection and generalist intervention based on common factors. The programme offers an alternative frontline treatment framework oriented towards PD to compensate for the lack of specialist PD services for young people. It can be viewed as a counterpart to GPM‐A (Choi‐Kain and Sharp 2021; Ilagan and Choi‐Kain 2021), which is an adaptation of GPM for adolescents with borderline BPD. Like GITPD‐A, it offers a generalist, common factors framework for treating young people with personality pathology. However, GPM perhaps leans more explicitly towards the categorical model of BPD than GITPD‐A, which is more rooted in new dimensional models.

To the best of our knowledge, no study has yet investigated the efficacy of a manualized generalist treatment for adolescents with developing PD. Therefore, a well‐controlled study, such as a randomized clinical trial, could be valuable. Such a study could demonstrate the efficacy of a structured, generalist, PD‐oriented treatment on relevant potential outcomes, including symptoms, stress, personality problems, quality of life and psychosocial functioning. The current study was a preparatory feasibility study for such a trial. It assessed the feasibility of the GITPD‐A intervention and a multicentre research design. Specifically, we assessed the intervention's acceptability among young people, considering treatment acceptability (based on Jørgensen et al. 2021, the intervention was considered acceptable if fewer than 35% of participants dropped out) and client satisfaction (we expected client satisfaction to be at least 7/10). We also estimated potential treatment benefits following the intervention (based on Cristea et al. 2017, meta‐analysis for BPD in adults, we expected an effect size of *d* = 0.70 to demonstrate the suitability of the selected primary outcome for assessing within‐subject changes between baseline and end‐of‐treatment). The feasibility of the research design was assessed by monitoring the recruitment of potential adolescents in each setting. Based on previous studies by Beck et al. (2020) and Schuppert et al. (2012), we expected that at least 50% of eligible young people would participate. Additionally, we monitored the retention of adolescents throughout the study. Based on the Dutch study by Schuppert et al. (2012), we expected that at least 70% of all participants would complete all measures. The information derived from this study was intended to inform the design of a larger‐scale randomized controlled study, including the sample size and preferred primary outcomes.

## 2 Methods

### 2.1 Study Design

The feasibility of the intervention and of a multicentre research design was studied using a one‐group pretest–posttest design, which is common in feasibility studies to evaluate the effectiveness of a new intervention (Bowen et al. 2009).

### 2.2 Participants

The participants were adolescents, aged 12–18, showing (emerging) PDs. They were recruited after undergoing a thorough screening process to determine their eligibility for both the treatment and the study. Specifically, adolescents were considered eligible for the study if they met the threshold for PDs according to the alternative model in the DSM‐5, meaning they had moderate or severe impairments in personality functioning (score ≥ 2), as assessed by the Semi‐Structured Interview for Personality Functioning‐DSM‐5 (STiP‐5.1) (Hutsebaut et al. 2017). Exclusion criteria included insufficient mastery of the Dutch language and cognitive impairment (IQ < 75). The WAIS‐IV was administered when intellectual impairment was suspected. Patients were also excluded if they met the DSM‐5 criteria for severe psychotic or substance abuse disorders, which required specific intervention.

### 2.3 Intervention

GITPD‐A is a generic treatment approach based on common factors of evidence‐based treatment approaches for PDs (Hutsebaut et al. 2020; Hutsebaut 2025). This manualized treatment framework addresses personality impairments and includes interventions that ensure common factors, such as building an alliance with young people and their parents, repairing potential ruptures, working towards shared decisions and responsibilities regarding treatment goals and tasks, enhancing self‐reflection and addressing (painful) emotions. GITPD‐A uses an enhanced common factor approach, including strategies for teams and institutions, to ensure appropriate early detection and intervention and improve team functioning. The average treatment duration is about 26 weeks (weekly sessions), with specific targets addressed in each phase. In the first phase (‘assessment phase’), careful assessment takes place, followed by a discussion of core problems and determination of the treatment focus by the therapist and patient. This phase focuses on shared decision‐making and examines the adolescent's needs, commitment to treatment, motivation for change and potential issues that could affect collaboration. The second phase (‘the treatment phase’) includes psychoeducation, crisis anticipation and implementation of selected treatment interventions focused on different personality impairments (e.g., emotion dysregulation and social cognition). The third phase prepares for the end of treatment and empowers young people and their families. This phase includes ‘booster’ sessions, which are usually less frequent than in the treatment phase. It is typical for GIT‐PD that the specific contents of treatment differ according to local traditions and competencies, but they should follow the common factor approach and cover relevant problem areas. A copy of the unpublished manual can be obtained from the last author.

All practitioners attended a 2‐day training course on the basic principles of GITPD‐A. In addition, treatment teams had access to the treatment manual and a series of model videos demonstrating the key common factors. To support implementation, a supervision structure was established, involving team leaders exchanging experiences approximately four times a year.

### 2.4 Procedure

The feasibility study took place in five mental health care institutions in the Netherlands with different backgrounds (Altrecht, Utrecht; IHuB, Amsterdam; Jeugd GGz, Zwolle; Karakter, Nijmegen; and de Viersprong, Bergen op Zoom). Some institutions (e.g., Altrecht) receive a wide variety of referrals, whereas others (e.g., de Viersprong) have a more specialist background. In line with the shift towards dimensional conceptualisations of personality pathology, eligibility was assessed using the Alternative Model for Personality Disorders (AMPD) framework. In most institutions, an initial screening procedure involving the Level of Personality Functioning Scale—Brief Form 2.0 (LPFS‐BF 2.0; Weekers et al. 2018; Wu et al. 2024) was carried out. The Semi‐Structured Interview for DSM‐5 Personality Functioning (STiP‐5.1; Hutsebaut et al. 2017) was then administered to adolescents displaying significant personality issues if they scored 26 or higher on the LPFS‐BF 2.0 (based on the normative data reported by Weekers et al. 2023). In other institutions, the STiP‐5.1 was used immediately to determine eligibility. Patients were considered eligible if they were assessed as having moderate or severe impairments in personality functioning, as determined by the interview rating. After the assessment, the mental healthcare professionals involved informed the eligible patients and their parents about the study. The patients and their parents received information leaflets about the study, and a member of the study team was available to answer questions. Patients were given a minimum of 1 week to decide whether to participate. If the patient (and their parents, if the patient was under 16 years old) agreed to participate and did not meet the exclusion criteria, they were asked to sign an informed consent form after the diagnostic procedure. After providing informed consent, participants completed the baseline measurements, consisting of the LPFS‐BF 2.0, PID‐5‐BF, GAD‐7, PHQ‐9, MSI‐BPD, DERS‐18, C‐SSRS and Kidscreen‐10 (see below), after which they started treatment. Measurements were conducted at baseline (T0) and at the end of treatment (T1). Treatment dropouts were also recorded. At the end of treatment, participants completed the self‐report questionnaires again, including the PID‐5‐BF, GAD‐7, PHQ‐9, MSI‐BPD, DERS‐18, C‐SSRS and Kidscreen‐10. Client satisfaction was assessed at the end of treatment using the ‘GGZ Client Thermometer’.

### 2.5 Measures

#### 2.5.1 Screening Measures

To screen for eligibility, the Semi‐structured Interview for Personality Functioning DSM‐5 (Hutsebaut et al. 2017) was used. The STiP‐5.1 is an interview schedule based on the DSM‐5 AMPD and measures the level of personality functioning in the following domains: Identity, Self‐Direction, Empathy and Intimacy. The interview consists of 28 open questions, with optional clarifying questions. Presence and overall severity of personality pathology were measured by delineating five levels of impairment of personality functioning, ranging from little or no impairment (Level 0) to extreme impairment of personality functioning (Level 4). Hutsebaut et al. (2017) demonstrated good to excellent interrater reliability of the interview‐based ratings in their study. Internal consistency and associations between domains and elements were remarkably high. The interview also showed good psychometric qualities in adolescents (Grigaitė et al. 2026; Thomson et al. 2024; Weekers et al. 2021). All of the clinicians who administered the interview had received online training in STIP, and they had access to supervision throughout the study.

#### 2.5.2 Feasibility Measures

The *feasibility* of the intervention was assessed using the following outcomes: Number of dropouts (%) during treatment, defined as a premature termination of treatment without mutual consent; Client satisfaction, as measured through the Jeugd GGz Thermometer, a widely used measure of client satisfaction. The total score of the ‘report grade’ and the mean of the four domains were calculated for ‘information’ (mean of Items 1–3), ‘participation’ (mean of Items 4–6), ‘therapist’ (mean of Items 7–10) and ‘result therapy’ (mean of Items 11–16). The feasibility of the multicentre research design was assessed using the following outcomes: total number of recruitment rates (%) defined as proportions/rates; total number of eligible adolescents (%) who consented to participate defined as proportions/rates; number of study dropouts (%) defined as proportions/rates, defined as refusal to continue participation in the data collection, regardless of treatment dropout; and missing data (%).

#### 2.5.3 Primary Outcome Measure

Preliminary *effectiveness* and feasibility of the selected primary outcome measure were studied using the Personality Inventory for DSM‐5 Brief Form: The PID‐5‐BF is an abbreviated 25‐item version (Krueger et al. 2013) of the original 220‐item PID‐5 (Krueger et al. 2012), designed to measure personality traits based on the AMPD. Each item is rated on a 4‐point Likert scale ranging from 0 (*very false or often false*) to 3 (*very true or often true*), with higher scores indicating greater dysfunction. The total score was calculated by summing the 25 items. Several studies have demonstrated acceptable reliability and validity of the PID‐5‐BF in adolescents (Anderson et al. 2018; Fossati et al. 2017; Venema et al. 2021; Zhang et al. 2021). Although the PID‐5‐BF is usually interpreted at the domain level, previous research has also used a total score as an overall index of maladaptive personality traits, including research in adolescent samples (e.g., Fossati et al. 2017). In the present study, the total score was used as a potential overall outcome measure to assess within‐person change in maladaptive personality traits. The PID‐5‐BF was selected as a potential primary outcome measure.

#### 2.5.4 Secondary Outcome Measures

(1) The Patient Health Questionnaire‐9 (PHQ‐9; Kroenke et al. 2001) was used to assess the severity of depressive symptoms. Depressive symptoms were included as a relevant secondary outcome because they commonly co‐occur with personality pathology and may change during treatment. The PHQ‐9 was selected due to its widespread use in routine outcome monitoring. The PHQ‐9 is a self‐administered instrument that assesses the frequency of nine symptoms of depression (i.e., mood, concentration, sleep and energy problems; low self‐esteem; and anhedonia) in the last 2 weeks on a 4‐point scale ranging from 0 (*not at all*) to 3 (*nearly every day*). The responses of each item were summed to provide a single score, ranging from 0 to 27, with higher scores indicating more severe symptoms of a depression. In our study, we used the total score that demonstrated high internal consistency in previous research (Johnson et al. 2002; Tsai et al. 2014; Zuckerbrot et al. 2018). (2) The Generalized Anxiety Disorder‐7 (GAD‐7; Spitzer et al. 2006) is a self‐report screening tool for generalized anxiety symptoms. Anxiety symptoms commonly co‐occur with personality pathology and may be relevant to changes associated with treatment. The scale consists of 7 items and reflects the frequency of symptoms during the preceding 2‐week period. The participant was requested to rate each item on a 4‐point Likert scale from 0 (*not at all*) to 3 (*nearly every day*). In this study, we calculated the total score, which provided an indication of the overall level of anxiety experienced by the patient. Several studies examined the psychometric properties of the questionnaire among adolescents and demonstrated good internal consistency, Cronbach's *α* = 0.91 (Mossman et al. 2017; Tiirikainen et al. 2019). (3) Kidscreen‐10 (Ravens‐Sieberer 2006) was used to measure the health‐related quality of life (HRQoL). HRQoL was included as a patient‐reported outcome to capture the broader impact of personality pathology and treatment on adolescents' everyday functioning and well‐being beyond changes in specific symptoms of psychopathology. This was considered particularly relevant because changes in personality functioning can have a significant impact on adolescents' overall well‐being and daily lives. This child self‐report questionnaire includes physical, emotional, social and cognitive functioning. The time frame refers to the past week. This brief instrument contains 10 items, and each item is answered on a 5‐point response scale. Answer categories for Items 1 and 9 are *not at all*–*slightly*–*moderately*–*very extremely*. The other items can be rated from *never*–*seldom*–*quite often*–*very often*–*always*. The total score of all 10 items was calculated to assess HRQoL. A higher total score indicated a higher level of HRQoL. The Kidscreen‐10 is a valid measure of HRQoL, and several studies found appropriate internal consistency (Ravens‐Sieberer et al. 2010; Stevanovic et al. 2013). (4) The McLean Screening Instrument for Borderline Personality Disorder (MSI‐BPD; Zanarini et al. 2003; Dutch translation by Schuppert et al. 2007) was used to assess BPD symptoms. The MSI‐BPD was included to complement the dimensional assessment of personality pathology by providing a measure that reflects the traditional categorical approach to PDs. Although the present study used the AMPD to evaluate personality functioning and dimensional personality pathology, symptoms of BPD remain clinically relevant in adolescents with emerging personality pathology. Including the MSI‐BPD therefore enabled us to examine changes in BPD‐specific symptoms alongside changes in broader personality functioning and maladaptive personality traits. The self‐report questionnaire consists of 10 dichotomous items corresponding to the DSM criteria for BPD, with a score of 7 or higher indicating the clinical cut‐off (Zanarini et al. 2003). In our study, we calculated the total score by summing the number of ‘yes’ responses, resulting in a score ranging from 0 to 10. Previous research has demonstrated adequate internal consistency of the Dutch version of the MSI‐BPD (*α* = 0.76; Verschuere and Tibboel 2011). The MSI‐BPD was therefore included as a secondary outcome measure to assess change in BPD‐specific symptomatology. (5) Difficulties in Emotion Regulation Scale‐18 (DERS‐18; Victor and Klonsky 2016) was included to assess difficulties in emotion regulation, which are particularly relevant to personality pathology and were therefore included as an important secondary outcome. The selection of the DERS‐18 was also informed by the ICHOM Standard Set for Personality Disorders, which includes difficulties in emotion regulation as an important outcome domain and recommends the DERS as an outcome measure (International Consortium for Health Outcomes Measurement, n.d.). The DERS‐18 consists of 18 items and has six subscales, each consisting of three items taken from the original DERS (Gratz and Roemer 2004). Items can be scored on six subscales: Non‐acceptance of Emotional Responses (Non‐acceptance); Difficulties Engaging in Goal‐Directed Behaviour (Goals); Lack of Emotional Awareness (Awareness); Limited Access to Emotion Regulation Strategies (Strategies); Impulse Control Difficulties (Impulse); and Lack of Emotional Clarity (Clarity). Each item was scored on a Likert scale ranging from 1 (*almost never*) to 5 (*almost always*). The total sum score of the 18 items was calculated, with higher scores reflecting greater difficulties in emotion regulation. The psychometric properties of the DERS‐18 have been studied in several studies. The DERS‐18 has strong internal consistency and validity in a community sample of adolescents (Charak et al. 2019; Victor and Klonsky 2016). (6) Columbia Suicide Severity Rating Scale (C‐SSRS; Posner et al. 2009) was used to assess the severity of suicidal ideation (e.g., a specific plan or method) and suicidal behaviour, including suicidal intent. The C‐SSRS was selected because suicidal thoughts and behaviour are significant outcomes for adolescents with PDs, and because the C‐SSRS is included in the ICHOM Set of Patient‐Centered Outcome Measures for Personality Disorders. ICHOM also recommends the C‐SSRS for assessing suicidal thoughts and behaviour in children and young people aged 10 years and over who present with anxiety and/or depression. The screener version consists of 3–6 ‘yes/no’ items, with the number of questions depending on the participant's responses. Adolescents are asked to indicate whether they have experienced suicidal thoughts or feelings during the past month and suicidal behaviour during the past 3 months and over their lifetime. Suicidal ideation is indicated by a ‘yes’ response to the initial questions regarding a wish to be dead and actual thoughts of killing oneself. If both questions are answered affirmatively, the participant is asked to complete the remaining questions. For this study, frequencies (i.e., percentages) at pre‐ and post‐measurement were reported. According to Posner et al. (2011), the C‐SSRS demonstrates good convergent and divergent validity, as well as high sensitivity and specificity for the classification of suicidal behaviour. Both the ideation and behaviour subscales have also demonstrated sensitivity to change over time.

### 2.6 Data Analyses

As this is a feasibility study, the primary goal was to estimate key parameters for a future main study, not to determine if there were statistically significant differences in the current study. Therefore, a formal a priori power calculation was not conducted. All data were entered into and analysed using SPSS version 30.0 (IBM Corp., Armonk, NY). Given this study's feasibility objectives, the outcome results were reported primarily descriptively, i.e., by reporting means and standard deviations, frequencies and percentages. Demographic data such as gender, age, education level, residential status, diagnosis and pharmacological treatments were collected prior to treatment. Reasons for dropping out and missing data values were also reported.

For the quantitative analysis of the primary and secondary outcomes within groups, the data were inspected for the following assumptions: outliers, normality, homogeneity, interval data and independence. No violations of these assumptions were observed in this study. To investigate the differences between the pre‐ and post‐assessments of the intended primary outcome (PID‐5‐BF) and the secondary outcomes (GAD‐7, PHQ‐9, MSI‐BPD, DERS‐18 and Kidscreen‐10), we used paired‐sample *t*‐tests. We used an alpha level of 0.05 for all statistical tests. To determine the feasibility of a larger, controlled main trial in the future, we computed the effect size of the intervention on the following outcome measures: PID‐5‐BF, GAD‐7, PHQ‐9, MSI‐BPD, DERS‐18 and Kidscreen‐10. Effect sizes were measured using Hedges' *g* and interpreted as follows: small = 0.2, medium = 0.5 and large = 0.8 (Cohen 1988).

## 3 Results

### 3.1 Feasibility of the Intervention

#### 3.1.1 Acceptability

Overall, 47 adolescents participated in the study. The distribution across institutions was as follows: Altrecht, *n* = 20 (42.6%); Jeugd GGz, *n* = 8 (17.0%); Karakter, *n* = 5 (10.6%); Opvoedpoli, *n* = 13 (27.7%); and Viersprong GGz, *n* = 1 (2.1%).

The treatment dropout rate was 19.1% (*n* = 9), below the benchmark of 35%. Figure 1 shows a consort‐style patient flow chart. Table 1 shows socio‐demographic characteristics of participating adolescents at baseline. Most of the adolescents were female (91.9%), and the mean age was 15.9 (SD = 1.3) years, with a range of 13–18.

**FIGURE 1:**
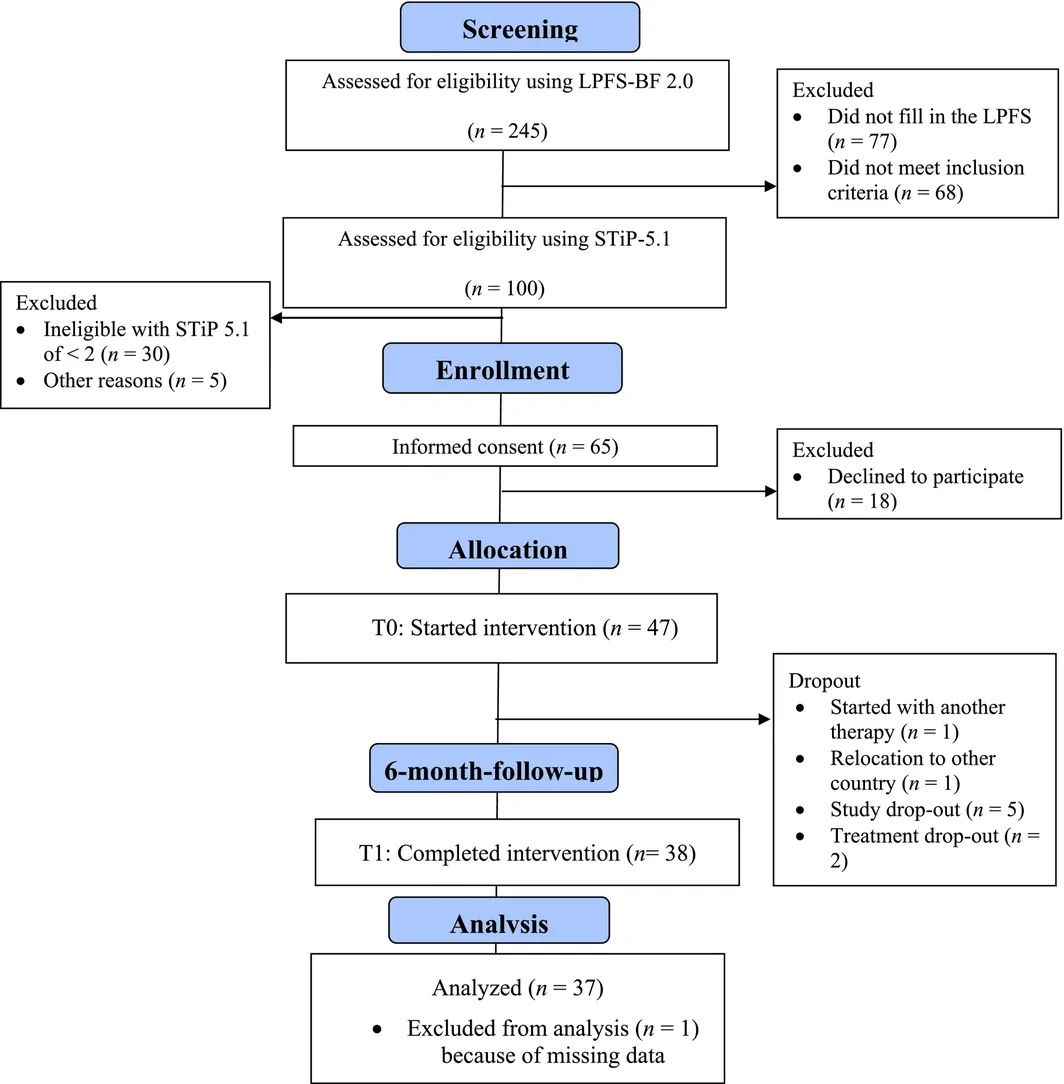
CONSORT flowchart of adolescents.

**TABLE 1:** Socio‐demographic characteristics of adolescents at baseline.

	*N*	%	
Gender			
Female	34	91.9	
Male	3	8.1	
Living situation			
Complete family	17	45.9	
Divorced parents	18	48.6	
Foster family	1	2.7	
Mental health crisis support	1	2.7	
Education (current)			
Secondary education—Vmbo	12	32.4	
Secondary education—Havo	12	32.4	
Secondary education—VWO	6	16.2	
Secondary vocational education (MBO)	5	13.5	
Other^a^	2	5.4	
Primary diagnosis			
Depressive disorders	2	5.4	
Anxiety disorders	1	2.7	
Personality disorders	23	62.2	
Developmental disorders	5	13.5	
Trauma‐ and stressor‐related disorders	5	13.5	
Missing	1	2.7	
Medication			
Antipsychotics	3	8.1	
Benzodiazepines	0	0	
Antidepressants	5	13.5	
Methylphenidate	7	18.9	
Other medication	5	13.5	
Treatment history	26	70.3	
Crisis admissions	1	2.7	
Screening instruments	*M*	SD	Range (min–max)
LPFS‐BF 2.0 Total functioning^b^	30.8	5.5	25 (14–39)
LPFS‐BF 2.0 Self‐functioning^b^	17.9	3.5	17 (6–23)
LPFS‐BF 2.0 Interpersonal functioning^b^	12.9	2.8	11 (6–17)
STiP‐5.1 Total score	2.09	0.56	1–3
STiP‐5.1 Identity	2.6	0.59	1–4
STiP‐5.1 Self‐direction	2.1	0.65	0–3
STiP‐5.1 Empathy	1.8	0.78	0–3
STiP‐5.1 Intimacy	1.9	0.87	0–4

^a^ Two adolescents attended special secondary education.

^b^ The total score of 33 adolescents was calculated because the scores of four adolescents were missing.

#### 3.1.2 Satisfaction With Treatment

Some organizations only had access to the Client Satisfaction Thermometer at a later time. This resulted in eight adolescents (24.3%) being unable to complete the questionnaire because their treatment had already ended. Twenty‐nine adolescents (78.4%) were given the Client Satisfaction Thermometer, and 28 adolescents (96.6%) completed it. On a scale from one to ten, 25 (89.3%) adolescents rated a seven or higher for overall satisfaction with the intervention (mean: 8.1), with five (13.5%) adolescents rating the intervention a 10. One (3.6%) adolescent was not satisfied with the treatment plan and would not recommend the intervention to others. This adolescent scored the total treatment a five. Most of the adolescents thought GITPD‐A is a good therapy for their mental issues, and they felt heard by their therapist. The provided information about the content of the intervention before the start was deemed ‘enough’ by 27 (96.4%) adolescents. Also, 23 (82.1%) adolescents reported being better able to deal with their problems and with other people compared with baseline. Four adolescents (14.3%) mentioned that they did not receive enough information about what they could expect from the outcome of the treatment. Regarding the results of the therapy, five adolescents (17.9%) did not feel they had enough control over their problems and symptoms after therapy, and eight adolescents (28.6%) reported being unable to do the things they wanted in life.

#### 3.1.3 Treatment Benefits

Table 2 shows the observed changes and effect sizes for the intended primary and secondary outcomes. The primary outcome (PID‐5) showed a significant difference in the total mean scores from baseline to post‐treatment with a medium effect size, demonstrating a decrease in pathological traits after the intervention. All intended secondary outcomes also showed significant decreases with medium effect sizes for severity of depression and anxiety, emotion dysregulation difficulties (assessed by DERS‐18) and BPD symptoms. However, none of the questionnaires demonstrated an effect size exceeding the benchmark. At baseline (T0), more than half of the adolescents (67.6%) reported active suicidal thoughts, and 26 (70.3%) adolescents reported suicidal ideation with a method. Approximately one quarter of the adolescents (32.4%) reported at least one lifetime suicide attempt, with the same proportion reporting an attempt within the past 3 months. Following treatment, 21 (58.3%) adolescents continued to report suicidal thoughts or behaviours; however, suicidal ideation with intent and plan was substantially reduced (5.9%). At post‐treatment (T1), two (5.4%) adolescents discontinued the questionnaire midway. The reasons for withdrawal were unclear.

**TABLE 2:** Secondary outcomes.

Variable	T0, pretest		T1, posttest					
	*M*	SD	*M*	SD	95% CI	*t*(36)	*p*s	Hedges' *g*
Primary outcome								
Personality Inventory for DSM‐5 Brief Form	36.35	9.63	32.16	13.67	1.43–6.95	3.08	**0.004**	0.50
Secondary outcomes								
Patient Health Questionnaire‐9^a^	16.9	5.2	13.8	6.1	0.84–5.22	2.81	**0.008**	0.46
Generalized Anxiety Disorder‐7^b^	13.5	4.8	10.8	5.5	1.02–4.26	3.31	**0.002**	0.54
Kidscreen‐10^c^	27.7	5.6	31.9	5.8	−6.22 to −2.32	−4.45	**< 0.001**	−0.72
McLean Screening Instrument for Borderline Personality Disorder^b^	7.7	2.4	6.5	3.0	0.43–2.01	3.15	**0.003**	0.51
Difficulties in Emotion Regulation Scale‐18^b^	65.3	11.2	57.5	14.5	5.94–18.28	4.14	0.**001**	0.57
Awareness	9.0	2.9	8.6	3.4	−0.81 to 1.37	0.54	0.308	0.17
Clarity	9.9	3.2	8.7	3.7	−1.41 to 3.42	0.87	0.123	0.26
Goals	13.1	2.4	12.3	2.4	1.01–3.10	4.15	0.092	0.28
Impulse	12.0	3.4	9.1	4.2	2.06–6.27	4.18	**< 0.001**	0.73
Nonacceptance	9.8	3.3	9.1	4.2	−0.43 to 3.32	1.62	0.325	0.16
Strategies	11.6	3.7	9.7	4.0	1.28–5.07	3.54	0.**008**	0.46
Columbia Suicide Severity Rating Scale	*n*	%	*n*	%				
Wish to be dead	25	67.6	21	58.3				
Suicidal thoughts	26	70.3	17	47.2				
SI with method, without plan or intent	22	59.5	11	32.4				
SI with intent, without plan	15	41.7	8	23.5				
SI with intent, with plan	7	19.4	2	5.9				
History with suicidal attempts	12	32.4	6	16.7				
In the past 3 months	12	32.4	7	20.0				

*Note: N* = 37. Significant differences are bolded. SI indicates suicidal ideation.

^a^ = 35.

^b^ = 36.

^c^ Higher scores indicate better HRQoL.

### 3.2 Feasibility of the Design

#### 3.2.1 Feasibility of Patient Recruitment

The flow chart of study adolescents is presented in Figure 1. In total, we recruited *n* = 245 adolescents with emerging PD symptoms; 168 of them (68.6%) were screened for eligibility with the LPFS‐BF 2.0, of whom 59.5% (*n* = 100) adolescents met the criteria to administer the STiP‐5.1 (total score > 26). Of these adolescents, 30 (30%) did not meet the eligibility criteria on the STiP‐5.1 (a score of 2 on at least 2 out of 4 elements), and five (5%) adolescents were excluded without a clear reason. After the screening procedure, a total of 65 adolescents were subsequently invited to provide informed consent. In total, 47 adolescents (72.3%) agreed to participate in the study, which is above the benchmark. Although exact reasons for declining participation in the study were not systematically recorded, the project coordinators from the five mental health institutions did provide general feedback on the reasons. Anecdotally, the most common reason for non‐participation in the study was lack of interest. One reason for the lack of interest in participating was that some adolescents found the study process too lengthy, as it required filling out several standardized questionnaires at the beginning and end of the treatment period. For some, this process felt too intrusive and tiring.

#### 3.2.2 Study Retention

Across the five participating mental health institutions, a total of 47 adolescents participated in the study at baseline. A total of 38 adolescents (80.1%) completed the intervention. The scores of one adolescent were excluded from the analyses. This adolescent consistently responded using two answer options instead of one, which rendered the questionnaires invalid for analysis. Except for a few missing items, all questionnaires were included in the analyses.

## 4 Discussion

This study aimed to explore the feasibility of a generalist intervention (GITPD‐A) for adolescents with emerging PD, as well as the feasibility of a multicentre study design, in preparation for a larger intervention study. Preliminary findings support the feasibility of the intervention, demonstrating low dropout rates, high satisfaction and moderate to substantial treatment benefits. The multicentre study design appears feasible, with high retention rates and sensitivity to change for the selected outcome measures. However, problems with study recruitment emerged, and questions were raised about the choice of the primary outcome measure. We will highlight and discuss some of the major findings.

### 4.1 Feasibility of the Intervention

Most adolescents who were eligible and agreed to participate remained engaged in treatment, resulting in a dropout rate of 19.1%. This rate is relatively low compared with those reported in recent studies on adolescents with BPD, which range from 15% to 75% (Jørgensen et al. 2021; Wong et al. 2020). Jørgensen et al. (2021) found that 45% of adolescents receiving mentalization‐based group therapy dropped out, and lower reflective functioning predicted dropout. Approximately half of the adolescents in the present study exhibited severe personality impairments indicative of low reflective capacities; however, they tended to persist in treatment in GITPD‐A. Although the reason for the higher retention in GITPD‐A is unclear, several factors may contribute to better engagement. Specialist programmes often involve intensive, highly structured interventions that may overwhelm adolescents emotionally (Andersen et al. 2021). Generalist programmes like GITPD‐A are less intensive and more tailored to the needs of young people and their families, which may support retention. Our data show that participants were well‐informed, highly satisfied with the treatment, trusted the therapists and valued the flexible, modular format that combined individual and family sessions. The focus on establishing authentic contact and maintaining a transparent, ‘human’ stance, rather than relying on methodical interventions, may have fostered the alliance (Hutsebaut 2025). This alliance may be vital for keeping adolescents in treatment (Cirasola et al. 2024). However, further research is needed to confirm whether low dropout rates are being replicated and to establish whether a personalized approach could help young people to stick with their treatment.

The overall observed treatment effects are medium in size. These results generally support the feasibility of the intervention. The magnitude of the effects is comparable with that of specialist interventions for adolescents with emerging BPD (Jørgensen et al. 2021; Wong et al. 2020). However, the observed effects are slightly smaller than those of comparable interventions for adults (Cristea et al. 2017). This is consistent with the finding that psychotherapy generally demonstrates smaller treatment benefits for children and adolescents (Cuijpers et al. 2019). Some argue that the observed treatment benefits in youth PD interventions reflect both curative and preventive effects (Kaess et al. 2024). When treatment begins early, features of PD psychopathology may not yet be fully established, and the benefits of treatment could also lie in preventing a further increase in PD symptoms. However, such preventive effects are not captured by the effect sizes. In hindsight, the chosen benchmark of 0.70 may be too high, and a future study should be adequately powered to detect relevant differences.

### 4.2 Feasibility of the Design

In general, *recruitment* for the study was problematic. Although a large number of young people were screened using the LPFS‐BF 2.0, only a small proportion (41%) were eligible for further screening with the STiP‐5.1 interview. Although this reflects the different approaches to implementing the screening procedure within the various institutions and their different patient samples, it also raises questions about the effectiveness of the LPFS‐BF 2.0 cut‐off point for identifying personality issues in adolescents. Although the LPFS‐BF 2.0 has undergone psychometric investigation in samples of adolescents (Wu et al. 2024), the cut‐off score of 26 used in the present study was derived from adult normative data (Weekers et al. 2023) and has not been specifically validated for use with adolescents. Consequently, using this cut‐off point may have meant that some adolescents with clinically relevant personality difficulties were not identified as eligible to participate. Furthermore, although many adolescents were eligible for further screening through the STiP‐5.1 interview, few were actually interviewed and included in the study. This may reflect the slow implementation of the AMPD in several institutions. Although the decision to use the AMPD to select participants was innovative and ensured that the study would be future‐proof, it also necessitated an additional implementation process, as clinicians had to be trained in the new model and its assessment. Furthermore, the feasibility study began at the same time as the treatment programme was implemented. More implementation resources were invested in clinical practice than in research. In hindsight, the large number of simultaneous implementation processes may have affected the study. Finally, we identified significant differences between institutions. Some institutions recruited and screened many more participants than others. This was partly due to personnel issues within institutions, such as staff absences due to illness or pregnancy, or the departure of a project coordinator. However, it was also due to the extent to which the institutions had embedded the research within the clinical pathway. Such implementation issues emphasize the importance of institutional and organizational processes, as well as potential patient characteristics. Indeed, a study of over 3000 patients across four mental healthcare institutions revealed significant variations in compliance with routine outcome monitoring, ranging from 8% to 60% (Trauer 2004). This variation was primarily influenced by institutional features and the integration of ROM logistics within individual treatment plans rather than patient characteristics. These findings suggest that mental healthcare institutions require additional support to incorporate the study design into their standard treatment protocols.

The findings of this study could inform the *selection of outcome measures* in future research. Although this study revealed a significant decrease in PID‐5 scores between the pre‐ and post‐measurements, the magnitude of the change was below our established threshold. This calls into question whether the PID‐5 is the most suitable primary outcome measure. The PID‐5 was selected because it is a well‐established instrument that measures maladaptive personality traits according to the AMPD. It has also been suggested that the total PID‐5 score indicates the severity of general personality pathology (Krueger et al. 2012). However, our results suggest that other aspects of personality pathology, such as emotion and impulse regulation, may capture changes in young people more sensitively. There may be several explanations. Firstly, emerging personality pathology has primarily been shown to manifest as emotional and impulse dysregulation in young people (Debast et al. 2017). Therefore, difficulties in emotional and impulse regulation may be relevant to many adolescents and could lead to significant therapeutic improvement. Secondly, the PID‐5 relates to Criterion B of the AMPD, which refers to the stylistic expression of impairments in personality functioning. However, one should consider maladaptive traits as potential expressions of these impairments. Consequently, only certain maladaptive traits will likely be elevated in individual cases, thereby restricting the range of possible improvements. Furthermore, Criterion B is believed to capture temperament‐related aspects of personality pathology that are biologically based and therefore less susceptible to change. In contrast, Criterion A, which refers to personality functioning, was designed to capture changeable aspects of personality pathology. A recent study demonstrated that treatment can more easily induce changes in personality functioning than in personality traits, particularly in vulnerable patients (Kiel et al. 2024). The authors concluded that personality functioning may be more sensitive to change than personality traits. Therefore, future studies considering changes in personality pathology as the most relevant outcomes could choose measures of personality functioning, particularly self‐functioning, as primary outcomes.

## 5 Limitations

The findings of this study should be considered in light of its limitations and the directions in which future research should proceed. These limitations include the relatively small sample size, which was predominantly female adolescents. This may affect how generalizable the results are. However, it should be noted that the study was designed to assess feasibility, and therefore, the results should not be considered as conclusions about the effectiveness of an intervention. Additionally, the context and timing of the measurements may have influenced the adolescents' responses and affected the effect size between the pre‐ and post‐treatment measurements. Research has shown that observed effect sizes are highly susceptible to the time point chosen for analysis (van Geffen et al. 2025). This is particularly pertinent given the use of self‐report questionnaires to assess outcomes. Furthermore, only the young people themselves were used as informants, and information on satisfaction or potential results was not obtained from parents. Finally, the current study did not include people with lived experience, either as part of the intervention or the study design. Therefore, we believe that the intended larger study should include a control condition, larger samples and interview measures in addition to self‐report measures. It should also involve people with lived experience in the design process.

## 6 Conclusion

The primary aim of GITPD‐A is to prevent personality problems from significantly impacting adolescent development. To achieve this, the programme designs an early detection pathway and a generalist intervention based on common factors of evidence‐based treatments. This study found preliminary support for the GITPD‐A intervention, which was positively valued by young people with emerging personality pathology. Regarding the feasibility of a multicentre study design, our results showed high retention rates, good acceptance of the intervention and sufficient ability to detect changes resulting from it. However, the current feasibility study also revealed significant challenges in recruiting participants, mainly related to a combination of implementation issues. Additional steps are necessary to recruit patients and establish a feasible multicentre randomized controlled trial to make well‐founded statements about the intervention's effect on a larger sample size.

## Funding

The authors have nothing to report.

## Ethics Statement

The study was preregistered on 15 April 2022 in the ISRCTN registry (ISRCTN41581). The study protocol was reviewed and approved by the Medical Research Ethics Committee Brabant (METC Brabant), the Netherlands (NL81304.028.22/P2220). The study was conducted in accordance with the applicable ethical and regulatory requirements for research involving human participants.

## Conflicts of Interest

The authors declare no conflicts of interest.

## Data Availability

The data that support the findings of this study are available from the corresponding author upon reasonable request.
